# Prevalence, predictors, and natural history of hypophosphatemia following iron infusion

**DOI:** 10.1093/jbmrpl/ziaf014

**Published:** 2025-12-06

**Authors:** Chloe Dawson, Lai-Ming Kathleen Pak, Eldho Paul, Naomi Szwarcbard, Kathryn Hackman

**Affiliations:** Department of Endocrinology and Diabetes, Alfred Health, Melbourne, VIC 3004, Australia; School of Public Health and Preventive Medicine, Monash University, Melbourne, VIC 3004, Australia; Department of Endocrinology and Diabetes, Alfred Health, Melbourne, VIC 3004, Australia; School of Public Health and Preventive Medicine, Monash University, Melbourne, VIC 3004, Australia; Department of Endocrinology and Diabetes, Alfred Health, Melbourne, VIC 3004, Australia; Department of Endocrinology and Diabetes, Alfred Health, Melbourne, VIC 3004, Australia; Department of Medicine, Monash University, Melbourne, VIC 3800, Australia

**Keywords:** hypophosphatemia, iron infusion, ferric carboxymaltose, iron polymaltose, ferric derisomaltose

## Abstract

Iron deficiency is the leading cause of anemia, which affects 1 quarter of the adult population globally. Intravenous iron is a well-established treatment option and is generally well tolerated. However, there is increasing recognition that it can cause hypophosphatemia, which can result in fatigue, nausea, myalgia, weakness, and osteomalacia. We sought to determine the prevalence, natural history, and predictors of hypophosphatemia following iron infusion in a tertiary hospital. All adult patients who received 1 or more iron infusions between January 1, 2019 and December 31, 2021 were included in this retrospective study. A total of 2367 subjects were identified, receiving a total of 7063 infusions over the study period. Hypophosphatemia was defined as a serum phosphate level of <0.75 mmol/L (<2.32 mg/dL), with severity classified as mild 0.65-0.75 mmol/L (2.01-2.31 mg/dL), moderate 0.32-0.64 mmol/L (0.99-1.98 mg/dL), or severe <0.32 mmol/L (<0.99 mg/dL). Prevalence of hypophosphatemia was 48.9%, with the highest prevalence from day 4 to 20 post-iron infusion (39.3%-44.1%). Risk factors were receipt of iron polymaltose (odds ratio [OR] 2.33, CI 1.71-3.19, *p* < .0001), receipt of ferric carboxymaltose (OR 1.79, CI 1.29-2.48, *p* = .001), male gender (OR 1.35, CI 1.09-1.66, *p* = .006), and multiple iron infusions (OR 1.56, CI 1.20-2.03, *p* = .001). Risk was reduced in recipients with higher baseline phosphate (OR 0.18, CI 0.11-0.28, *p* < .0001), higher baseline creatinine (OR 0.83, CI 0.74-0.92, *p* = .001), and increased weight (OR 0.99, CI 0.99-1.00, *p* = .016). Risk factors for moderate to severe hypophosphatemia were the same, except that gender became nonsignificant, while increasing age (OR 0.99, CI 0.99-1.00, *p* = .002) was also associated with reduced risk. These findings may aid clinicians in identifying those at greatest risk of developing significant hypophosphatemia, thereby preventing adverse outcomes.

## Graphical Abstract

**Figure:**
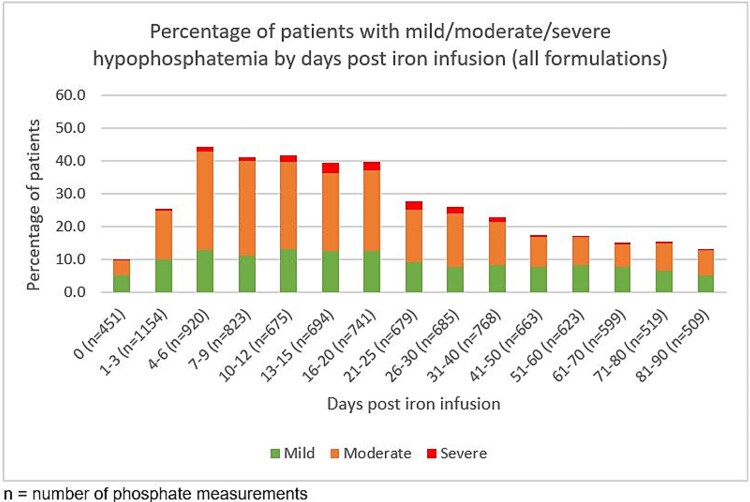

## Introduction

Anemia affects 24.3% of the world’s adult population, with iron deficiency the leading cause.^1^ Its prevalence is higher in those with poor dietary intake, heavy menstrual bleeds, chronic kidney disease, gastrointestinal bleeding, and malabsorption syndromes. Oral iron supplementation is first-line treatment but has poor bioavailability and can result in significant gastrointestinal side effects.^2^ A single infusion of intravenous (IV) iron can provide up to 2500 mg of elemental iron and is indicated when oral therapy is ineffective or not tolerated. There are 4 IV iron preparations available in Australia. The 2 most commonly prescribed are iron polymaltose (introduced in 2002)^3^ and ferric carboxymaltose (introduced in 2011).^4^ Iron sucrose (introduced in 2004)^5^ is mainly available for use in hemodialysis patients and ferric derisomaltose, also known as iron isomaltoside, is a newer agent (introduced in 2017).^6^ Ferric carboxymaltose is preferred in the ambulatory setting as it is given as a 15-min infusion, however maximum dose per infusion is 1000 mg of elemental iron. In contrast, iron polymaltose is more commonly used in the inpatient setting, and provides up to 2500 mg of elemental iron, but can take up to 5 h per infusion.^7^ Although hypophosphatemia is described as a “common” side effect of ferric carboxymaltose^4^ and “infrequent” of iron polymaltose^3^ in the Australian product information, in clinical practice, this side effect is not widely recognized by healthcare professionals. Further, hypophosphatemia following iron polymaltose has not been well studied, possibly because its use is mainly confined to Australia and New Zealand.^8^

Hypophosphatemia post-iron infusion has been reported to result in fatigue,^9^^,^^10^ dizziness,^10^^,^^11^ nausea,^10^^,^^12^ muscle pain, and weakness.^10^^,^^12^^,^^13^ There have been case reports of hypophosphatemic osteomalacia and bone deformities in patients receiving repeated doses of ferric carboxymaltose.^14^^–^^17^ Severe cases of hypophosphatemia can lead to rhabdomyolysis, altered mental state, seizures, congestive heart failure, and respiratory depression.^18^ There have been case reports of altered mental state,^19^^,^^20^ respiratory depression,^21^^,^^22^ and seizures^23^ associated with hypophosphatemia post ferric carboxymaltose infusion.

Iron deficiency is associated with increased levels of cleaved, biologically inactive FGF23, but normal levels of biologically active intact FGF23.^24^ The underlying cause of hypophosphatemia following iron infusion relates to the carbohydrate moieties within IV iron preparations, which prevent the degradation/cleavage of FGF23, resulting in an increase in intact FGF23 levels.^24^ FGF23, secreted by osteoblasts and osetocytes, is a phosphaturic hormone that reduces phosphate reabsorption in the renal proximal tubule in response to raised serum phosphate levels.^25^^,^^26^ It also reduces production of 1,25-OH vitamin D (activated vitamin D) via suppression of 1-α hydroxylase activity in the kidneys, resulting in reduced intestinal calcium absorption and increased renal calcium excretion.^25^ Low activated vitamin D and hypocalcemia cause secondary hyperparathyroidism, which further contributes to phosphaturia and hypophosphatemia.^24^^,^^27^ This compensatory hyperparathyroidism can further prolong renal phosphate wasting, even after FGF23 levels have normalized.^28^ The incidence of hypophosphatemia following iron infusion is poorly defined. In a 2020 systematic review of United States marketed IV iron preparations, the incidence of hypophosphatemia varied widely, ranging from 0%-92% for ferric carboxymaltose, 0%-40% for iron sucrose, 0.4% for ferumoxytol, and 0% for low molecular weight iron dextran.^29^ A concurrent European systematic review of the 2 iron preparations available for rapid infusion, which included most of the same randomized control trials (RCTs), also reported a hypophosphatemia prevalence of 0%-92%, and demonstrated pooled hypophosphatemia rates of 47% following ferric carboxymaltose and 4% following ferric derisomaltose infusion.^30^ There is limited data investigating the incidence of iron polymaltose-associated hypophosphatemia. A single-center 8 participant prospective study demonstrated a decrease in mean phosphate level from 3.4 ± 0.6 to 1.8 ± 0.6 mg/dL (1.1 ± 0.2 to 0.6 ± 0.2 mmol/L) 1 wk after iron polymaltose infusion (ranging from 500 to 1600 mg elemental iron). In this cohort, plasma phosphate levels normalized by 7 wk.^31^

The above studies assessed the overall incidence of hypophosphatemia; however they did not discriminate between levels of severity, which may be more clinically relevant. Retrospective studies^9^^,^^32^ as well as RCTs^33^^,^^34^ that have differentiated levels of severity of hypophosphatemia only included ferric carboxymaltose, iron sucrose, and ferumoxytol formulations. Few studies have investigated the natural history of hypophosphatemia over a prolonged period including its time of onset and time to resolution. While a secondary analysis from the PHOSPHARE-IDA trial investigated the severity and duration of hypophosphatemia following ferric carboxymaltose and ferric derisomaltose, patient numbers were small and follow-up was for a maximum of 35 d post-infusion.^35^ Risk factors for hypophosphatemia are also inconsistent across studies.

Given the variability of the current literature, we sought to determine the prevalence of iron-induced hypophosphatemia, the time course for its development and resolution (natural history), and risk factors for its development in a contemporary Australian cohort. We also stratified hypophosphatemia into clinical categories of mild, moderate, and severe.

## Materials and methods

### Study design

This was a retrospective study of all inpatients and outpatients aged ≥18 yr who received an iron infusion at The Alfred Hospital in Melbourne, Victoria, Australia, between January 1, 2019 and December 31, 2021. All patients who had a phosphate measurement within the 3 mo following iron infusion were included in the study.

### Data collection

Data obtained from the electronic medical records included (1) patient demographics (age, sex, weight), (2) biochemistry results (phosphate, calcium, magnesium, creatinine, vitamin D, PTH, hemoglobin, ferritin), (3) date, type (formulation) of iron infusion, and number of infusions received from 3 mo prior to study commencement until study end, and (4) date of any IV phosphate replacement within 3 mo post-infusion. All relevant pathology investigations from 3 mo pre- to 6 mo post-iron infusion were collected. The baseline biochemistry was defined as that prior to and closest to the date of infusion within the preceding 3 mo of the infusion. Data were de-identified and securely stored.

### Iron formulations

The 4 types of IV iron administered at our institution over the study period were iron polymaltose, ferric carboxymaltose, ferric derisomaltose, and iron sucrose. Standard dose per infusion for these preparations are iron polymaltose 1000-2000 mg of elemental iron, ferric carboxymaltose 500-1000 mg of elemental iron, ferric derisomaltose 1500 mg of elemental iron, and iron sucrose 500 mg of elemental iron. The choice of iron infusion type administered was at the discretion of the clinician prescribing the infusion.

### Phosphate measurement

Phosphate levels were measured on an Abbott Alinity instrument using a phosphomolybdate method with the analysis reliability shown in Table S1. The measurement of uncertainty was not recorded for the year 2020 due to COVID disruptions.

### Hypophosphatemia classification

Hypophosphatemia was defined as a serum phosphate level of <0.75 mmol/L (<2.32 mg/dL). Severity was classified as mild 0.65-0.75 mmol/L (2.01-2.31 mg/dL), moderate 0.32-0.64 mmol/L (0.99-1.98 mg/dL), or severe <0.32 mmol/L (<0.99 mg/dL). Where phosphate values were reported as <0.23 or < 0.21, these values were arbitrarily assigned a numerical value of 0.20 for inclusion in the analysis.

### Statistical analysis

 Continuous variables were presented using means and SDs or medians and IQRs according to data type and distribution. The natural history of hypophosphatemia following iron infusion was determined by calculating the percentage of patients with normal, mild, moderate, and severely low phosphate on grouped days 0, 1-3, 4-6, 7-9, 10-12, 13-15, 16-20, 21-25, 26-30, 31-40, 41-50, 51-60, 61-70, 71-80, and 81-90, using the lowest phosphate measurement per patient per time period. All phosphate measurements from the first recorded iron infusion until subsequent infusions or 90 d (whichever came first) were included. As the number of patients experiencing hypophosphatemia decreased over time, we increased the grouped day interval for ease of interpretation (Table S2).

Change in mean phosphate levels pre- and post-infusion was determined by calculating the mean phosphate level at baseline (using the measurement taken closest to date of infusion within the 3 mo prior) and comparing it with the mean nadir phosphate post-infusion over 90-d follow-up. Only patients who had baseline phosphate measurements were included in this calculation.

To determine the risk factors for developing hypophosphatemia post iron infusion, patients were initially categorized into those who developed hypophosphatemia at any time in the 3 mo following their initial iron infusion and those who did not. Hypophosphatemia was defined as a phosphate level < 0.75 mmol/L. We then determined the risk factors for more clinically significant hypophosphatemia, by comparing those with normal and mild hypophosphatemia (phosphate level ≥ 0.65 mmol/L) to those with moderate and severe hypophosphatemia (phosphate level < 0.65 mmol/L). The risk of hypophosphatemia was determined based on the first iron formulation received during the study period.

Univariate and multivariate analysis were performed to determine risk factors for hypophosphatemia overall and moderate or severe hypophosphatemia using logistic regression, with results reported as odds ratios (ORs) and 95% CIs. Variables with a *p* < .05 on univariate analysis and those deemed clinically relevant were included in the multivariate models. The variables considered for inclusion in multivariate models were age, sex, weight, phosphate, creatinine, calcium, magnesium, type of iron infusion, and number of iron infusions. Estimated glomerular filtration rate (eGFR) was not included in the multivariate analysis due to collinearity with the variables weight and creatinine. PTH and iron sucrose were not included in the multivariate analysis due to low numbers. All *p* values were 2-sided with a *p* value of <.05 considered statistically significant. Analyses were performed with SAS software version 9.4 (SAS Institute, Cary, NC, United States).

Approval to conduct the research was granted by The Alfred Ethics Committee (288/21). Procedural informed consent was obtained from all patients prior to receiving an iron infusion as per local protocols at the Alfred Hospital.

## Results

### Patient characteristics

A total of 4756 adults received 9774 iron infusions at the Alfred Hospital in Melbourne, Australia, between January 1, 2019 and December 31, 2021. Of these subjects, 2389 were excluded due to not having a serum phosphate measurement recorded within 3 mo post iron infusion. The remaining 2367 adults included in the study received 7063 iron infusions. The majority received iron polymaltose, *n* = 4990 (70.6%), followed by ferric carboxymaltose, *n* = 1320 (18.7%), ferric derisomaltose, *n* = 541 (7.7%), and iron sucrose, *n* = 212 (3.0%). Most initial iron infusions were iron polymaltose, *n* = 1093 (46.2%) and ferric carboxymaltose, *n* = 918 (38.8%), followed by ferric derisomaltose, *n* = 341 (14.4%), and iron sucrose, *n* = 15 (0.6%). Most patients received a single iron infusion, *n* = 1725 (72.9%). Of the 642 (27.1%) who received more than 1 infusion, the median number of infusions was 2 (IQR 2-6).

Phosphate was measured in 74.1% of recipients (*n* = 1755) prior to infusion. In the 3 mo following infusion, 29 500 serum phosphate measurements were recorded in 2367 recipients. Patient characteristics and baseline biochemistry are shown in Table 1.

**Table 1:** Subject characteristics.

**Subject characteristics (*n* = 2367)**	
**Age (yr), mean ± SD (*n* = 2367)**	65.5 ± 18.7
**Female sex, n (%)** **Male sex, n (%)**	1153 (48.7) 1214 (51.3)
**Weight (kg), mean ± SD (*n* = 2332)**	74.8 ± 21.4
**Type of initial iron infusion** **Iron polymaltose, n (%)** **Ferric carboxymaltose, n (%)** **Ferric derisomaltose, n (%)** **Iron sucrose, n (%)**	1093 (46.2) 918 (38.8) 341 (14.4) 15 (0.6)
**Number of iron infusions per patient** **1 infusion, n (%)** **2 infusions, n (%)** **3 infusions, n (%)** **≥4 infusions, n (%)**	1725 (72.9) 328 (13.9) 81 (3.4) 233 (9.8)
**Total number of iron infusions (*n* = 7063)** **Iron polymaltose, n (%)** **Ferric carboxymaltose, n (%)** **Ferric derisomaltose, n (%)** **Iron sucrose, n (%)**	4990 (70.6) 1320 (18.7) 541 (7.7) 212 (3.0)
**Baseline creatinine (RR: 45-90 umol/L), median [IQR] (*n* = 1919)**	86 [66-135]
**Baseline eGFR (RR: >90 mL/min/1.73m^2^), median [IQR] (*n* = 1914)**	68 [40-91]
**Baseline phosphate (RR: 0.75-1.50 mmol/L), mean ± SD (*n* = 1755)**	1.12 ± 0.33
**Baseline phosphate (mmol/L), (*n* = 1755)** **Normal ≥0.75, n (%)** **Mild low 0.65-0.74, n (%)** **Moderate low 0.32-0.64, n (%)** **Severe low <0.32, n (%)**	1631 (92.9) 75 (4.3) 48 (2.7) 1 (0.1)
**Baseline calcium (RR: 2.10-2.60 mmol/L), mean ± SD (*n* = 1756)**	2.24 ± 0.16
**Baseline magnesium (RR: 0.70-1.10 mmol/L), mean ± SD (*n* = 1753)**	0.81 ± 0.13
**Baseline PTH (RR: 1.60-6.00 pmol/L), median [IQR] (*n* = 251)**	10 [5.4-20.5]
**Baseline ferritin (RR: 30-300 mcg/L), median [IQR] (*n* = 1801)**	44 [21-127]
**Baseline hemoglobin (RR: 115-155 g/L), mean ± SD (*n* = 1936)**	104.0 ± 21.4
**Baseline vitamin D (RR: >50 nmol/L), median [IQR] (*n* = 696)**	70 [43-96]

Abbreviations: eGFR, estimated glomerular filtration rate; RR, reference range for normal values.

### Primary outcomes

#### Prevalence of hypophosphatemia

Mean phosphate prior to infusion was 1.12 ± 0.33 mmol/L and mean nadir phosphate post-infusion was 0.79 ± 0.34 mmol/L. Mean difference was 0.33 (95% CI 0.31-0.35) mmol/L. Baseline prevalence of hypophosphatemia was 7.1% (124/1755). About 5.6% (99/1755) were hypophosphatemic both pre- and post-infusion. Of the patients who had hypophosphatemia pre-infusion, it was mild, moderate, and severe in 4.3% (*n* = 75), 2.7% (*n* = 48), and 0.1% (*n* = 1), respectively. Incidence of hypophosphatemia post-infusion was 43.5% (764/1755); which was mild, moderate, and severe in 12.7% (*n* = 223), 32.0% (*n* = 562), and 4.4% (*n* = 78) respectively. Post-iron infusion, the overall prevalence of hypophosphatemia was 48.9% (1158/2367). Prevalence of moderate or severe hypophosphatemia following infusion was 36.4%. Prevalence of hypophosphatemia was significantly higher in those who received multiple infusions, 54% (*n* = 347), compared with those who received a single infusion, 47% (*n* = 811) (*p* = .002).

#### Natural history of hypophosphatemia following iron infusion

**Figure 1:**
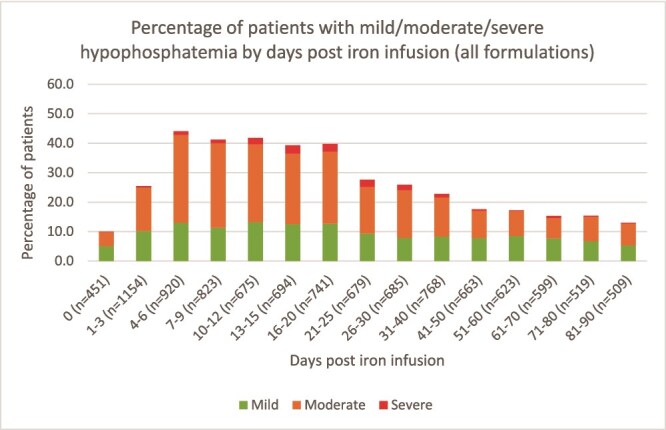
Timeline of hypophosphatemia over 3 mo post iron infusion; *n* is the number of phosphate measurements.

The percentage of patients with mild, moderate, and severe hypophosphatemia at each timepoint post iron infusion is shown in Figure 1. Prevalence of hypophosphatemia was highest from day 4 to day 20 post-iron infusion, ranging from 39.3% to 44.1%, and reduced thereafter.

One quarter of patients (26.8%) had only 1 timepoint of phosphate measurement following infusion. Most (63.1%) had measurements in 1-4 timepoints, and 10.5% had more than 10 timepoints (Table S2).

**Figure 2:**
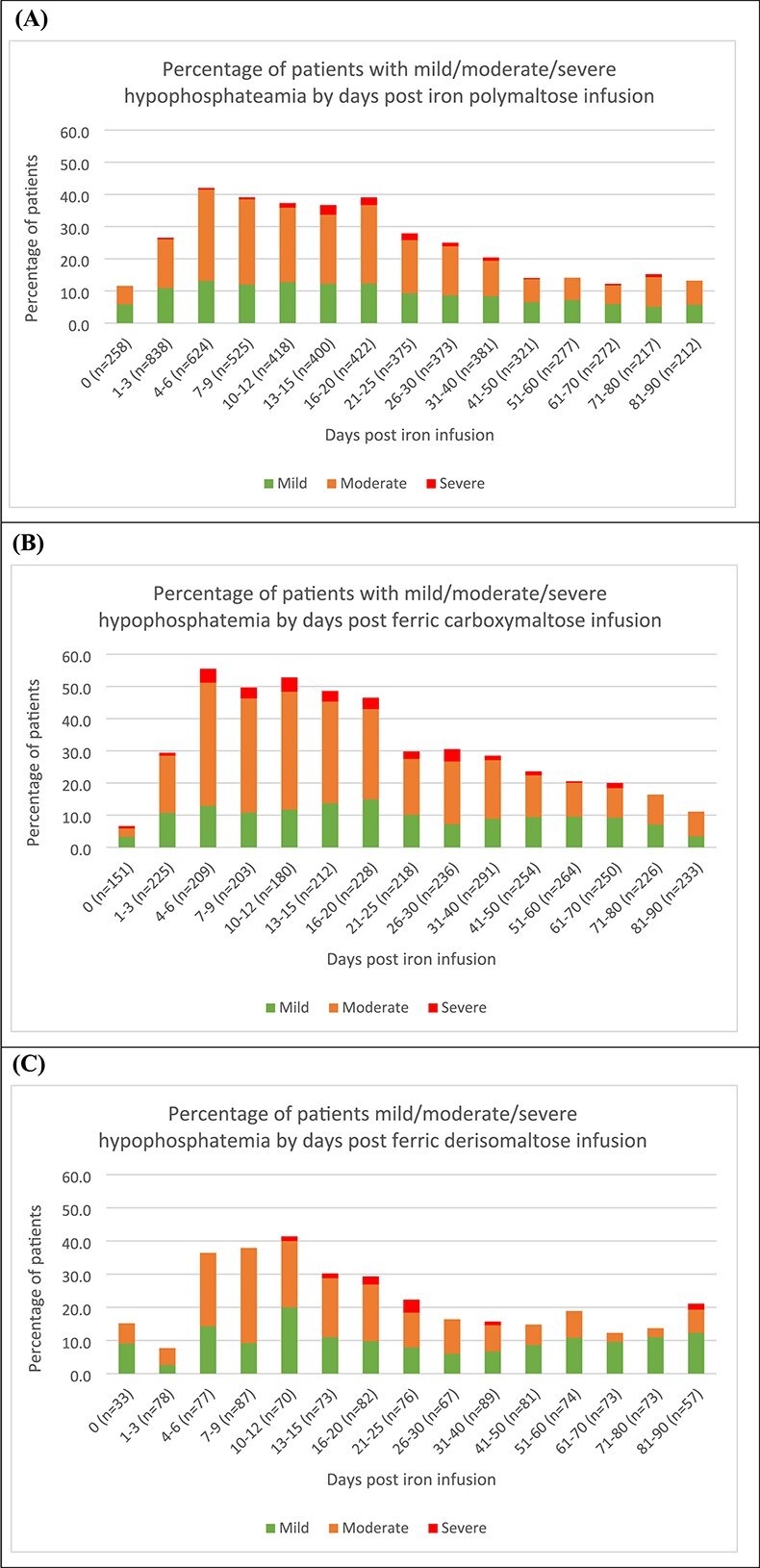
Timeline of hypophosphataemia over 3 mo post (A) iron polymaltose, (B) ferric carboxymaltose, and (C) ferric derisomaltose infusion; *n* is the number of phosphate measurements.

The percentage of patients with mild, moderate, and severe hypophosphatemia at each timepoint following iron polymaltose, ferric carboxymaltose, and ferric derisomaltose is shown in Figure 2. Prevalence of hypophosphatemia was highest from day 4 to day 20 for all infusions, and ranged from 36.7% to 42% for iron polymaltose, 46.5% to 55.5% for ferric carboxymaltose, and 29.3% to 41.4% for ferric derisomaltose. Prevalence of hypophosphatemia appeared highest on days 4-6 for both iron polymaltose and ferric carboxmaltose, and highest on days 10-12 for ferric derisomaltose.

#### Risk factors for hypophosphatemia following iron infusion

On univariate analyses, higher baseline eGFR (OR 1.14, 95% CI 1.10-1.18, *p* < .0001), iron polymaltose (OR 1.32, 95% CI 1.13-1.56, *p* = .001), and receipt of 2 iron infusions (OR 1.46, 95% CI 1.15-1.85, *p* = .002) were associated with increased risk of hypophosphatemia.

Risk of hypophosphatemia was reduced in those with increased weight (OR 0.99, 95% CI 0.99-1.00, *p* = .002), increased creatinine (OR 0.71, 95% CI 0.65-0.78, *p* < .0001), increased phosphate (OR 0.13, 95% CI 0.09-0.20, *p* < .0001), and increased magnesium (OR 0.38, 95% CI 0.18-0.81, *p* = .012) at baseline, use of ferric derisomaltose (OR 0.59, 95% CI 0.47-0.75, *p* < .0001), and receipt of a single iron infusion (OR 0.75, 95% CI 0.63-0.91, *p* = .002).

In patients with mild hypophosphatemia at baseline, the OR for hypophosphatemia post-infusion was 2.92 (95% CI 1.75-4.88, *p* < .0001), whereas those with moderate/severe hypophosphatemia at baseline had an OR of 12.77 (95% CI 4.57-35.66, *p* < .0001).

There was no association between use of ferric carboxymaltose, iron sucrose, baseline PTH, vitamin D levels, ferritin, hemoglobin, or receipt of more than 2 iron infusions and hypophosphatemia (Table S3).

On multivariate analysis (Table 2), independent risk factors for hypophosphatemia were receipt of iron polymaltose (OR 2.33, 95% CI 1.71-3.19, *p* < .0001) or ferric carboxymaltose (OR 1.79, 95% CI 1.29-2.48, *p* = .001) when compared with ferric derisomaltose, multiple infusions (OR 1.56, 95% CI 1.20-2.03, *p* = .001) and male sex (OR 1.35, 95% CI 1.09-1.66, *p* = .006). Risk of hypophosphatemia was reduced in those with higher baseline creatinine (OR 0.83, 95% CI 0.74-0.92, *p* = .001) and phosphate (OR 0.18, 95% CI 0.11-0.28, *p* < .0001). For each kilogram of weight increase, risk reduced by 1% (OR 0.99, 95% CI 0.99-1.00, *p* = .016).

**Table 2:** Multivariate analysis of risk factors for hypophosphatemia following iron infusion.

**Characteristics**	**Odds ratio for hypophosphatemia (95% CI)**	***p* value**
**Age (yr)**	1.00 (0.99-1.00)	.175
**Sex** **Female** **Male**	1.0 1.35 (1.09-1.66)	**.006**
**Weight (kg)**	0.99 (0.99-1.00)	**.016**
**Baseline phosphate (mmol/L)**	0.18 (0.11-0.28)	**<.0001**
**Baseline creatinine (umol/L)**	0.83 (0.74-0.92)	**.001**
**Baseline calcium (mmol/L)**	0.77 (0.40-1.49)	.435
**Baseline magnesium (mmol/L)**	0.95 (0.41-2.20)	.909
**Ferric carboxymaltose**	1.79 (1.29-2.48)	**.001**
**Iron polymaltose**	2.33 (1.71-3.19)	**<.0001**
**Multiple iron infusions**	1.56 (1.20-2.03)	**.001**

#### Risk factors for moderate or severe hypophosphatemia following iron infusion

On univariate analyses, receipt of only 1 infusion reduced risk of moderate to severe hypophosphatemia (OR 0.75, 95% CI 0.63-0.91, *p* = .003), 2 infusions increased risk (OR 1.42, 95% CI 1.12-1.80, *p* = .004), and there was no association with more than 2 infusions. Other risk factors for moderate or severe hypophosphatemia were younger age (OR 0.99, 95% CI 0.99-1.00, *p* = .012), lower weight (OR 0.99, 95% CI 0.99-1.00, *p* = .004), lower creatinine (OR 0.72, 95% CI 0.65-0.80, *p* < .0001), lower baseline phosphate (OR 0.20, 95% CI 0.14-0.30, *p* < .0001), and use of iron polymaltose (OR 1.33, 95% CI 1.13-1.57, *p* = .001). Use of ferric derisomaltose (OR 0.52, 95% CI 0.40-0.68, *p* < .0001) and iron sucrose (OR 0.12, 95% CI 0.02-0.94, *p* = .044) were associated with reduced risk (Table S4).

On multivariate analysis, risk factors for moderate or severe hypophosphatemia were the receipt of iron polymaltose (OR 2.24, 95% CI 1.60-3.14, *p* < .0001), ferric carboxymaltose (OR 1.76, 95% CI 1.24-2.51, *p* = .002), and multiple iron infusions (OR 1.48, 95% CI 1.14-1.92, *p* = .003). Increasing age (OR 0.99, 95% CI 0.99-1.00, *p* = .002), higher creatinine (OR 0.83, 95% CI 0.74-0.93, *p* = .001), and higher baseline phosphate (OR 0.26, 95% CI 0.17-0.41, *p* < .0001) were associated with reduced risk of moderate or severe hypophosphatemia (Table 3).

**Table 3:** Multivariate analysis of risk factors for moderate or severe hypophosphatemia following iron infusion.

**Characteristics**	**Odds ratio for moderate/severe hypophosphatemia (95% CI)**	***p* value**
**Age (yr)**	0.99 (0.99-1.00)	**.002**
**Sex** **Female** **Male**	1.0 1.22 (0.98-1.51)	.071
**Weight (kg)**	1.00 (0.99-1.00)	.073
**Baseline phosphate (mmol/L)**	0.26 (0.17-0.41)	**<.0001**
**Baseline creatinine (umol/L)**	0.83 (0.74-0.93)	**.001**
**Baseline calcium (mmol/L)**	0.69 (0.35-1.34)	.274
**Baseline magnesium (mmol/L)**	1.09 (0.46-2.57)	.840
**Ferric carboxymaltose**	1.76 (1.24-2.51)	**.002**
**Iron polymaltose**	2.24 (1.60-3.14)	**<.0001**
**Multiple iron infusions**	1.48 (1.14-1.92)	**.003**

#### Treatment with IV phosphate replacement

A total of 18 patients received IV phosphate replacement, of whom 16 had hypophosphatemia and 2 had normal phosphate post iron infusion. Of the 104 patients (4.4%) who had severe hypophosphatemia, only 1 patient received IV phosphate replacement.

## Discussion

In the largest study to date to assess hypophosphatemia following iron infusion, we report an incidence of 43.5%, and a prevalence of 48.9%. Baseline prevalence of hypophosphatemia prior to iron infusion was 7.1%, which is similar to previous retrospective studies.^36^^,^^37^ Prevalence of moderate or severe hypophosphatemia was 36.4%. Hypophosphatemia appeared to occur more frequently in the first 4-20 d following infusion, and gradually decreased thereafter. Risk of hypophosphatemia was highest in those who received iron polymaltose and ferric carboxymaltose, and those who had multiple infusions. Male sex, and lower creatinine, phosphate, and weight at baseline were also risk factors.

Although previous systematic reviews have reported hypophosphatemia prevalence of “up to” 92%, this figure comes from a prospective, single-arm study including only 38 patients, all of whom received ferric carboxymaltose as the sole iron formulation.^38^ Our finding of hypophosphatemia in nearly half of our iron infusion recipients was higher than anticipated, particularly given only 18.7% received ferric carboxymaltose.

The time course for development of hypophosphatemia in this study was similar to most previous smaller studies, which have reported a phosphate nadir at around 2 to 3 wk post iron infusion.^33^^,^^39^ A retrospective cohort study of 81 patients who received either ferric carboxymaltose or ferric derisomaltose reported nadir phosphate levels within the first 60 d after infusion.^32^ In the 17 patients who had longer follow-up, median time to normalization of phosphate was 84 d (IQR 62-185 d).^32^ However, a retrospective study of 130 patients reported a mean hypophosphatemic period of 6 mo in patients who received ferric carboxymaltose and a range of 2 to 18 wk for iron sucrose.^9^ Both these studies were limited by a small number of phosphate measurements and multiple subsequent infusions, the effect of which were not assessed. In this study, the prevalence of hypophosphatemia appeared greatest at days 4-6 with ferric carboxymaltose and iron polymaltose, and days 10-12 with ferric derisomaltose.

We found that ferric carboxymaltose was associated with a 79% increased risk of hypophosphatemia. This is consistent with systematic reviews and meta-analyses which have shown it to be associated with the highest risk and severity of hypophosphatemia when compared with iron sucrose, ferric derisomaltose, iron dextran and ferumoxytol.^29^^,^^30^ However, we also demonstrated that iron polymaltose has a similar risk of hypophosphatemia, with an OR of 2.33 (95% CI 1.71-3.19, *p* < .0001). Iron polymaltose was also associated with a more than 2-fold increased risk of moderate or severe hypophosphatemia. While Australian product information lists hypophosphatemia as a common adverse effect following ferric carboxymaltose, it is currently listed as “infrequent” for iron polymaltose.^3^ This is a novel finding of clinical significance. Our findings are consistent with previous studies, which show low or no risk of hypophosphatemia with ferric derisomaltose.^30^^,^^32^^,^^34^^,^^40^^,^^41^

Our findings of increased prevalence and earlier onset of hypophosphatemia with ferric carboxymaltose and iron polymaltose are in keeping with the proposed mechanism of hypophosphatemia being due to differences in the chemical structure of the bound carbohydrate moiety in the type of iron formulation given.^28^^,^^42^^,^^43^ Ferric carboxymaltose and iron polymaltose share similar carbohydrate moieties, while ferric derisomaltose forms a structurally different iron-carbohydrate complex.^44^ This may explain the similar findings for the first 2 formulations while the latter conferred no increased risk of hypophosphatemia. Unfortunately, at the time of this analysis, FGF23 testing was not available at our site.

Similar to previous studies, we showed that multiple iron infusions also increased risk of hypophosphatemia. This has been demonstrated with ferric carboxymaltose. Barish et al.^45^ randomized 1435 patients to single-dose (*n* = 366) and multi-dose (*n* = 343) ferric carboxymaltose, and single (*n* = 360) and multi-dose (*n* = 366) “standard medical care”, mostly with IV iron sucrose and iron dextran. They demonstrated hypophosphatemia in 47.8% of patients receiving multiple ferric carboxymaltose infusions compared with only 18% who received a single ferric carboxymaltose infusion. The incidence of hypophosphatemia in the standard care group remained 0.3%, irrespective of the number of doses received.^45^ A smaller study of 78 subjects found that cumulative doses of ferric carboxymaltose were associated with risk of hypophosphatemia.^9^ Repeated iron polymaltose infusion has also been shown to be associated with prolonged hypophosphatemia and subsequent insufficiency fractures in 1 case report.^46^ We also found an increased risk of hypophosphatemia in subjects who received multiple infusions, but were unable to further stratify this risk by iron formulation type or specific number of infusions administered.

Consistent with previous studies, we found that low phosphate,^32^^,^^33^ lower creatinine,^30^^,^^33^ and lower body weight^33^ prior to iron infusion were associated with development of hypophosphatemia. In contrast to the literature, we found an independent association between younger age and moderate or severe hypophosphatemia, as well as male sex and hypophosphatemia, which remained statistically significant after adjusting for renal function on multivariate analysis. Smaller studies have not found an association with age or sex.^9^ It has previously been shown that post-menopausal women typically have higher phosphate levels than men,^47^ which may explain our finding of an increased risk of hypophosphatemia in men compared with women. We did not find an association between baseline calcium, vitamin D, or PTH level in the development of hypophosphatemia. The literature regarding these associations is unclear, with 1 RCT in a group of 130 patients and 1 retrospective cohort study of 81 patients finding no association with vitamin D.^9^ However, 2 other randomized trials of a similar size found a decrease in 1,25-OH vitamin D levels and an increase in PTH following administration of ferric carboxymaltose, compared with ferric derisomaltose.^34^^,^^40^ These trials did not assess other risk factors for hypophosphatemia such as age and sex.

It is notable that despite severe hypophosphatemia in 104 patients, only 1 received IV phosphate replacement. This may reflect clinicians’ preference for oral supplementation, which we were unable to assess in this study, or a lack of awareness of the clinical implications of the condition. The requirement for IV phosphate replacement may represent a surrogate marker for the clinical significance of hypophosphatemia. IV replacement is associated with closer clinical and biochemical monitoring, higher cost, and possibly a prolonged hospital admission. While IV replacement is important to prevent acute and severe adverse effects from hypophosphatemia, it should be noted that due to the prolonged duration of elevated iFGF23 and renal phosphate wasting, phosphate replacement is not a practical long-term treatment for iron-induced hypophosphatemia. Future research may explore the clinical impact of hypophosphatemia, such as adverse medical outcomes, quality of life, and cost to the healthcare system. This may lead to development of protocols guiding the frequency and duration of serum phosphate measurement following iron infusion.

This study is the largest to date to assess the effect of different IV iron formulations on serum phosphate, and compared the most commonly used iron formulations available in clinical practice in Australia. It is the first to include iron polymaltose, which had previously been assessed in a sample of only 8 patients.^31^ This study is also the first to delineate, in detail, the time course of hypophosphatemia following iron infusion, and categorize it by severity in a large population. Iron infusions administered during the study period occurred in both inpatient and outpatient settings, therefore encompassing patients with multiple comorbidities and a variety of clinical acuity, thus increasing the generalizability of the patient cohort.

There were several limitations to this study. First, its retrospective design resulted in the timing of phosphate measurements not being standardized across the cohort. Second, there were relatively few measurements of PTH and vitamin D, which necessitated their omission from multivariate analysis. Third, we were unable to assess use of other medications that could affect serum phosphate, including receipt of oral phosphate supplementation. Fourth, we were also unable to assess the etiology of iron deficiency, which has been shown in previous studies to affect the risk of hypophosphatemia, in that patients with gastrointestinal or gynecological blood loss are more often reported to have symptomatic hypophosphatemia post-iron infusions than patients with chronic kidney disease.^28^

Finally, given the size of this study and volume of biochemistry data, only the formulation of the initial iron infusion within the study period was analyzed. However, it may not have been the patient’s first or only preparation of iron infusion received. This limits the generalizability of the findings to a single or initial iron infusion, and may cause confounding with patients who had previously received iron infusions prior to the commencement of the study.

Although it is possible that those with low phosphate levels had multiple measurements, resulting in bias, this risk was minimized by including only 1 phosphate measurement per patient per timepoint. Further, most patients had phosphate measured over up to 4 timepoints, with fewer than 10% having a phosphate measurement at more than ten timepoints. More than 500 individuals were included at each timepoint after the day of infusion.

## Conclusion

In this large, contemporary study of inpatients and outpatients receiving iron infusions, prevalence of hypophosphatemia was 48.9% over 90 d. Moderate to severe hypophosphatemia occurred in 36.4%. Risk was greatest with iron polymaltose and ferric carboxymaltose formulations. Multiple infusions, male sex, and lower creatinine further increased risk. These findings may aid clinicians in identifying those at greatest risk of developing significant hypophosphatemia and contribute both to prescribing choices and frequency of biochemical monitoring following iron infusion, thereby potentially preventing adverse clinical outcomes.

## Supplementary Material

Phosphate_Supplement_FINAL_ziaf014

## Data Availability

The data that support the findings of this study are available from the corresponding author upon reasonable request. The data are not publicly available due to patient privacy.
