# Supplementary material for: Prevalence, predictors, and natural history of hypophosphatemia following iron infusion
Source: JBMR Plus. 2025 Dec 6;9(Suppl 5):v69–78. doi: 10.1093/jbmrpl/ziaf014 (PMC12723802; doi:10.1093/jbmrpl/ziaf014)
Supplement: Phosphate_Supplement_FINAL_ziaf014 [file phosphate_supplement_final_ziaf014.docx]

**Supplemental Methodology Analysis**

We performed a preliminary analysis to test our hypothesis that there was unlikely to be a causative association between iron infusion and phosphate measurements taken more than 3 months later, and to determine whether we would have sufficient data to perform meaningful analyses if we excluded patients who did not have a phosphate measurement within 3 months of infusion. This included the proportion of patients who had phosphate measured within 3- and 6- months and who had hypophosphatemia within 1-, 3- and 6- months of infusion. Our analysis demonstrated that 67% of low phosphate measurements occurred in the first month, 89% had occurred within 3 months, and 93% had occurred within 6 months post single or multiple iron infusion/s. Furthermore, out of the total phosphate measurements (n = 32636) recorded 6 months post infusion, 91.4% of all measurements were performed within the first 3 months. Based on these findings we elected to exclude patients who did not have a phosphate measurement within 3 months of infusion.

**Supplemental Table 1. Phosphate analysis reliability**

| **Year** | **Phosphate Measurement of Uncertainty** | | | |
| --- | --- | --- | --- | --- |
|  | **Low Value** | **Coefficient of variation (%)** | **High Value** | **Coefficient of variation (%)** |
| 2019 | 1.01 | 1.7 | 2.38 | 1.3 |
| 2021 | 0.95 | 1.3 | 2.41 | 0.9 |

**Supplemental Table 2. Number of patients with phosphate measurements per time point**

| No. of time points | No. of patients |
| --- | --- |
| 1 | 634 |
| 2 | 357 |
| 3 | 291 |
| 4 | 212 |
| 5 | 171 |
| 6 | 131 |
| 7 | 97 |
| 8 | 96 |
| 9 | 73 |
| 10 | 56 |
| 11 | 68 |
| 12 | 60 |
| 13 | 50 |
| 14 | 54 |
| 15 | 17 |
| Total | 2367 |

**Supplemental Table 3. Univariate analysis of risk factors for hypophosphatemia following iron infusion**

| **Characteristics** | **Normal phosphate** | **Hypophosphatemia** | **Odds Ratio for hypophosphatemia (95% CI)** | **P value** |
| --- | --- | --- | --- | --- |
| Age (years), mean ± SD | 65.64 ± 19.12 | 65.26 ± 18.19 | 1.00 (0.99 – 1.00) | 0.620 |
| Sex  Female  Male | 49.3% (596)  50.7% (613) | 48.1% (557)  51.9% (601) | 1.0  1.05 (0.89 – 1.23) | 0.560 |
| Baseline weight (kg), mean ± SD | 76.17 ± 21.94 | 73.40 ± 20.79 | 0.99 (0.99 – 1.00) | **0.002** |
| Baseline phosphate (mmol/L), mean ± SD | 1.20 ± 0.37 | 1.03 ± 0.26 | 0.13 (0.09 – 0.20) | **<0.0001** |
| Baseline creatinine (umol/L), median [IQR] | 96.0 [67 – 157] | 81.0 [65 – 115] | 0.71 (0.65 – 0.78) | **<0.0001** |
| Baseline eGFR (mL/min/1.73m2), median [IQR] | 61 [31 – 90] | 73.5 [49 – 91] | 1.14 (1.10 – 1.18) | **<0.0001** |
| Baseline calcium (mmol/L), mean ± SD | 2.25 ± 0.16 | 2.24 ± 0.16 | 0.72 (0.40 – 1.29) | 0.264 |
| Baseline magnesium (mmol/L), mean ± SD | 0.82 ± 0.12 | 0.81 ± 0.13 | 0.38 (0.18 – 0.81) | **0.012** |
| Baseline PTH (pmol/L), median [IQR] | 13.3 [6.2 – 25] | 7.4 [4.4 – 17] | 0.99 (0.97 – 1.00) | 0.070 |
| Baseline vitamin D (nmol/L), median [IQR] | 70 [42 – 96] | 70 [45 – 97] | 1.00 (1.00 – 1.00) | 0.915 |
| Baseline ferritin (mcg/L), median [IQR] | 43 [21 – 127.5] | 45 [22 – 127] | 1.01 (0.98 – 1.05) | 0.452 |
| Baseline hemoglobin (g/L), mean ± SD | 103.46 ± 21.20 | 104.51 ± 21.67 | 1.00 (1.00 – 1.01) | 0.282 |
| Ferric carboxymaltose (%) | 469 (38.8%) | 449 (38.8%) | 1.00 (0.85 – 1.18) | 0.993 |
| Ferric derisomaltose (%) | 212 (17.5%) | 129 (11.1%) | 0.59 (0.47 – 0.75) | **<0.0001** |
| Iron polymaltose (%) | 517 (42.8%) | 576 (49.7%) | 1.32 (1.13 – 1.56) | **0.001** |
| Iron sucrose (%) | 11 (0.9%) | 4 (0.3%) | 0.38 (0.12 – 1.19) | 0.096 |
| Number of iron infusions per patient  1 infusion (%)  2 infusions (%)  3 infusions (%)  >3 infusions (%) | 914 (75.6%)  141 (11.7%)  38 (3.1%)  116 (9.6%) | 811 (70.0%) 187 (16.1%)  43 (3.7%)  117 (10.1%) | 0.75 (0.63 – 0.91)  1.46 (1.15 – 1.85)  1.19 (0.76 **–** 1.85)  1.06 (0.81 **–** 1.39) | **0.002**  **0.002**  0.446  0.678 |

**Supplemental Table 4. Univariate analysis of risk factors for moderate or severe hypophosphatemia following iron infusion**

| **Characteristics** | **Normal/mild hypophosphatemia** | **Moderate/severe hypophosphatemia** | **Odds Ratio for hypophosphatemia (CI 95%)** | **P value** |
| --- | --- | --- | --- | --- |
| Age (years), mean ± SD | 66.19 ± 18.83 | 64.18 ± 18.32 | 0.99 (0.99 – 1.00) | **0.012** |
| Sex  Female  Male | 48.7% (733)  51.3% (772) | 48.7% (420)  51.3% (442) | 1.0  1.00 (0.85 – 1.18) | 0.993 |
| Baseline weight (kg), mean ± SD | 75.77 ± 21.93 | 73.14 ± 20.43 | 0.99 (0.99 – 1.00) | **0.004** |
| Baseline phosphate (mmol/L), mean ± SD | 1.17 ± 0.35 | 1.03 ± 0.27 | 0.20 (0.14 – 0.30) | **<0.0001** |
| Baseline creatinine (umol/L), median [IQR] | 92 [67 – 148] | 78 [65 – 110] | 0.72 (0.65 – 0.80) | **<0.0001** |
| Baseline eGFR (mL/min/1.73m2), median [IQR] | 63 [35 – 89] | 76 [52 – 91] | 1.15 (1.11 – 1.19) | **<0.0001** |
| Baseline calcium (mmol/L), mean ± SD | 2.25 ± 0.16 | 2.23 ± 0.16 | 0.65 (0.35 – 1.19) | 0.162 |
| Baseline magnesium (mmol/L), mean ± SD | 0.82 ± 0.12 | 0.81 ± 0.13 | 0.46 (0.21-1.01) | 0.054 |
| Baseline PTH (pmol/L), median [IQR] | 11.7 [6.1 – 23.1] | 6.95 [4.4 – 15.2] | 0.99 (0.91 – 1.00) | 0.098 |
| Baseline vitamin D (nmol/L), median [IQR] | 69.5 [41 – 96.5] | 71.0 [46 – 94.5] | 1.00 (1.00 – 1.00) | 0.729 |
| Baseline ferritin (mcg/L), median [IQR] | 44.5 [22 – 124] | 44.0 [21 – 135] | 1.02 (0.98 – 1.05) | 0.368 |
| Baseline hemoglobin (g/L), mean ± SD | 103.52 ± 21.43 | 104.78 ± 21.42 | 1.00 (1.00 – 1.01) | 0.214 |
| Ferric carboxymaltose (%) | 578 (38.4%) | 340 (39.4%) | 1.04 (0.88 – 1.24) | 0.618 |
| Ferric derisomaltose | 257 (17.1%) | 84 (9.7%) | 0.52 (0.40 – 0.68) | **<0.0001** |
| Iron polymaltose | 656 (43.6%) | 437 (50.7%) | 1.33 (1.13 – 1.57) | **0.001** |
| Iron sucrose | 14 (0.9%) | 1 (0.1) | 0.12 (0.02 – 0.94) | **0.044** |
| Number of iron infusions per patient  1 infusion (%)  2 infusions (%)  3 infusions (%)  >3 infusions (%) | 1128 (75.0%)  185 (12.3%)  47 (3.1%)  145 (9.6%) | 597 (69.3%)  143 (16.6%)  34 (3.9%)  88 (10.2%) | 0.75 (0.63 – 0.91)  1.42 (1.12 – 1.80)  1.27 (0.81 – 2.00)  1.07 (0.81 – 1.41) | **0.003**  **0.004**  0.291  0.650 |
